# Pilot non dialysis chronic renal insufficiency study (P-ND-CRIS): a pilot study of an open prospective hospital-based French cohort

**DOI:** 10.1186/s12882-017-0463-3

**Published:** 2017-02-01

**Authors:** Jacques Massol, Gérard Janin, Camille Bachot, Christophe Gousset, Geoffroy Sainte-Claire Deville, Jean-Marc Chalopin

**Affiliations:** 1 Institut PHISQUARE, 20, rue Saint Saëns, 75015 Paris, France; 2 Centre Hospitalier de Mâcon, 350, boulevard Escande, 71018 Mâcon, Cedex France; 3 Nephrology, CHRU de Besançon, Hôpital Jean Minjoz, 3, boulevard Alexandre Fleming, 25030 Besançon, France

**Keywords:** Chronic renal disease, Renal insufficiency, Cohort, Epidemiology, Pilot study, Pharmacoepidemiology

## Abstract

**Background:**

Before establishing a prospective cohort, an initial pilot study is recommended. However, there are no precise guidelines on this subject.

This paper reports the findings of a French regional pilot study carried out in three nephrology departments, before realizing a major prospective Non Dialysis Chronic Renal Insufficiency study (ND-CRIS).

**Methods:**

We carried out an internal pilot study. The objectives of this pilot study were to validate the feasibility (regulatory approval, providing patients with information, availability of variables, refusal rate of eligible patients) and quality criteria (missing data, rate of patients lost to follow-up, characteristics of the patients included and non-included eligible patients, quality control of the data gathered) and estimate the human resources necessary (number of clinical research associates required).

**Results:**

The authorizations obtained (CCTIRS – CNIL) and the contracts signed with hospitals have fulfilled the regulatory requirements. After validating the information on the study provided to patients, 1849 of them were included in three centres (university hospital, intercommunal hospital, town hospital) between April 2012 and September 2015. The low refusal rate (51 patients) and the characteristics of non-included patients have confirmed the benefit for patients of participating in the study and provide evidence of the feasibility and representativeness of the population studied. The lack of missing data on the variables studied, the quality of the data analyzed and the low number of patients lost to follow-up are evidence of the quality of the study. By taking into account the time spent by CRAs to enter data and to travel, as well as the annual patient numbers in each hospital, we estimate that five CRAs will be required in total.

**Conclusion:**

With no specific guidelines on how to realize a pilot study before implementing a major prospective cohort, we considered it pertinent to report our experience of P-ND-CRIS. This experience confirms that i) feasibility, ii) quality of data and iii) evaluating the resources required must be validated before carrying out a large prospective cohort study such as ND-CRIS.

**Electronic supplementary material:**

The online version of this article (doi:10.1186/s12882-017-0463-3) contains supplementary material, which is available to authorized users.

## Background

The importance of conducting epidemiological cohorts in France has been highlighted on several occasions [1]. Chronic kidney disease (CKD) places a heavy burden on health services, and it is becoming increasingly common [2]. End-stage renal disease (ESRD) is only the tip of the CKD iceberg [3]. With early diagnosis and treatment, it is possible to slow the progression of kidney disease [3–5]. The prevalence in France of chronic renal insufficiency with GFR < 60 ml/mn/1.73 m^2^ has been estimated at 8.2% according to the Mona Lisa study in a French population of patients aged between 35 and 75 [6] and 12% in patients aged > 65 in the 3C Study [7].

There are few prospective cohort studies on CKD. There is no long-running epidemiological study on pre-dialysis CKD, and incident cohorts are also lacking in France [8].

This is why we set up the Non-Dialysis Chronic Renal Insufficiency Study (ND-CRIS), a prospective cohort of patients managed in the nephrology units of a French region (Bourgogne Franche-Comté).

The primary aim of the ND-CRIS cohort is to describe the ND-CRIS population followed by nephrologists in the Bourgogne-Franche-Comté region (eastern France). It is intended to provide regular information on therapeutic care and on the biological tests prescribed by physicians in the centres. The quality of the care provided is assessed using indicators based on those recommended by ANAES and HAS.

The ND-CRIS cohort is also designed to describe the outcomes of patients included until they require replacement therapies (dialysis, transplant, etc.). It also intends to describe outcomes in terms of the characteristics of this population. It thus provides information on epidemiological trends among patients with CKD in the Bourgogne-Franche-Comté region. This information will facilitate ad hoc studies assessing the risk-benefit ratio of health products and actions.

However, a cohort study, which requires considerable resources, cannot always be implemented for feasibility reasons, especially if it is undertaken in more than one hospital with no temporal horizon.

Before launching a study on this scale, a pilot study should be performed as recommended in the good practice guidelines for epidemiology [9]. However, unlike for clinical trials [10], there are no guidelines to our knowledge on how to conduct pilot cohort studies. Having carried out a pilot study in three of the nine nephrology departments in Bourgogne Franche-Comté, we believe our experience could assist other epidemiology researchers, particularly in the field of chronic diseases such as renal insufficiency.

The aim of this pilot study was to validate feasibility and the quality of the data, and estimate the resources required to extend the cohort to six other hospitals in the Bourgogne-Franche-Comté region.

## Methods

### Population

The population consisted of adult patients (>18 years) with chronic kidney disease (CKD) who had follow-up appointments with nephrologists in the hospitals of Mâcon, Belfort-Montbéliard and Besançon University Hospital.

The eligible population consisted of patients presenting with CKD with a GFR < 60 ml/min/1.73 m^2^, calculated using the MDRD method (stages 3 to 5: CKD stage defined as an estimated GFR of 45–59 (stage 3a), 30–44 (stage 3b), 15–29 (stage 4) and <15 (stage 5) ml/min/1.73 m^2^) and not receiving dialysis [11].

The population included patients presenting CKD with two measurements of GFR < 60 ml/min/1.73 m^2^ calculated with the MDRD method and not receiving dialysis.

### Methods

#### Aims

To validate feasibility, we intended to i: ensure that all hospitals were fully committed to the study before the pilot study was conducted in three hospitals (pre-test), ii: obtain regulatory approval and authorization at a national level (CCTIRS and CNIL) and at a regional level (hospitals), iii: undertake the process of providing patients with information (comprehension test and information leaflet), iiii: ensure that the variables of interest were present and accessible in the medical files.

To validate the quality of the data, we focused on i: analyzing characteristics of the patients included and non-included eligible patients and comparing these two groups to ensure the population studied was representative of this type of population, ii: measuring missing data and the rate of patients lost to follow-up, iii: checking the quality of data collected during appointments, enabling us to verify the data gathered by the CRAs.

The necessary resources (number of CRAs) were estimated based on the average time required for a CRA to enter patient data into the CRF, the time required by CRAs to travel to the participating hospitals, and the potential number of patients in each nephrology unit in the Bourgogne-Franche-Comté region.

#### Design

This was an internal pilot study on a prospective cohort with no predefined temporal horizon, i.e. the patients included and followed in the pilot study would be included in the ND-CRIS cohort, and their follow-up continued.

#### Duration

This pilot study was conducted between April 2012 and September 2015 in three of the nine hospitals in the region. The duration of three years was chosen because it was consistent with the frequency of follow-up consultations for patients with non-dialysis CKD (at least one consultation per year), as reported by the investigators.

#### Pre-test

Before the cohort was established, a pre-test phase was designed to ascertain the commitment of the management and nephrology teams in each hospital. It was done during face to face meetings in each centre of nephrology. The main objective of these meetings was to obtain insurance of their commitment. During these meetings, comments and remarks have been integrated into the protocol. No formal survey was necessary to collect feedbacks.

#### Pilot phase

The pilot study consisted of the inclusion phase and follow-up of patients. The CRAs were asked to search in medical files for the main variables concerned (age, sex, GFR, comorbidity – diabetes and hypertension and exposure to medication) in both inclusion appointments and follow-up consultations.

The present study was intended to refine the list of variables and to finalize the initial protocol before submitting it for approval by the regulatory authorities (CNIL and CCTIRS).

Meetings were held in each hospital to explain the cohort project, and the different groups of patients were evaluated during these meetings.

#### Inclusion/exclusion criteria

According to this protocol, which was accepted for publication [12], patients were included if they presented a GFR < 60 ml/min/1.73 m^2^, and if they did not refuse to participate in the study as required by the French legislation (see section “Ethics and Consent” of the Declarations).

We excluded patients who had received transplants, those who had undergone dialysis, those attending occasional appointments in the participating hospitals and patients who were unable to understand the information leaflet.

#### Definition of subjects lost to follow-up

The duration of the P-ND-CRIS cohort was adjusted according to the estimated mean frequency of follow-up consultations to obtain a group of patients included for at least 18 months. This enabled us to assess the potential attrition rate.

Although the cohort involved all patients with a GFR < 60 ml/mn/1.73 m^2^ managed in a nephrology unit, nephrologists do not see patients with a GFR > 45 ml/mn systematically (this is the threshold beyond which patients should be followed in nephrology departments according to the HAS [13]).

The majority of patients whose GFR was between 45 and 60 ml/mn/1.73 m^2^ are managed by general practitioners. These patients cannot therefore be considered as lost to follow up if they have not been seen for 18 months. We therefore defined patients lost to follow up as patients whose GFR was < 45 ml/mn/1.73 m^2^ and had not been seen for at least 18 months.

#### Data collection and data management

After obtaining access to medical files (in paper or electronic form) from the hospital management team, the Department of Medical information (DIM) and the physician in charge of the department, three CRAs identified eligible patients. The physicians then explained the study to eligible patients and reported to the CRAs to inform them of any refusals to take part. Information on patient refusals was noted on the consultation report or in the patient’s electronic data file. Data were gathered by the CRAs in the participating hospitals from medical files, using an electronic case-report form (CRF). The data were collected when the patient was included and at each follow-up consultation (or hospitalization). The data from each hospital were then transferred every three months to the data manager and stored in an SAS database.

#### Coordination and compliance with good epidemiological practice

The study as a whole was managed by a coordinating project leader, a steering committee and a manager.

The collection, processing and storing of data were performed in compliance with deontological ethics and good epidemiological practice recommendations (ADELF, ADEREST, AEEMA, EPITER) [French 2007 version 9] and with the ISPE Guidelines for good pharmacoepidemiological practice (GPP) [14].

#### Data collected

**Table:** 

Data collected	At screening	At inclusion	At follow-up consultations	When leaving the cohort
Age / gender/ area of residence / socio-professional status	X			
Date of consultation	X	x	X	
Creatininaemia	X	x	X	
Diagnosis of kidney disease		X		
Risk factors, complications and hospitalizations		x	X	
Clinical examination: weight, height, blood pressure		x	X	
Biological tests: proteinuria, microalbuminuria, calcaemia, phosphoremia, haemoglobin, 25 OHD3, PTH, ferritin, iron, saturation, CRF		x	x	
Examinations/imaging with injection of contrast substances			x	
Pharmacological treatment		x	x	
Date and type of information on dialysis, date and method of dialysis, date of fistula placement				x
Date and type of information on transplants. Date of transplant				x
Death and cause				x
Other reasons for removal from cohort (specify)				x

#### Quality control

At the end of the pilot phase, a quality assessment was conducted in the three hospitals in order to determine discrepancies between the variables listed in the CRFs and those obtained from the medical files. Discrepancies were classified as minor or major, according to criteria defined by the steering committee.

Minor discrepancies were defined as having no impact on patient characteristics (e.g. level of renal function deterioration, identification of comorbidities or administration of relevant medication). These minor discrepancies sometimes involved collecting more recent measurements from a medical file, an error in noting the result of a titration schedule with no consequences for the patient’s profile, the omission of a minor medication excluded from the list of indicators defining the protocol objectives, or failure to complete a variable in the CRF when that variable was not related to the main cohort objectives.

Major discrepancies were defined as having an impact on the main patient characteristics. These include a follow-up consultation omitted from the CRF, or a comorbidity not noted in the CRF (failure to collect data in a follow-up consultation; non-identification of cardiac failure as a relevant comorbidity, non-identification of ACE Inhibitor as a relevant treatment in the table of treatments).

#### Statistical analyses

The patients included were described for all three hospitals and for each hospital individually. The quantitative variables were described using whole numbers, the number of elements of data entered, means and standard deviation, and extreme (min/max) values. The statistical tests were two-tailed and the statistical significance threshold was set at 5%. The statistical analyses were performed on SAS® software, version 9.4, SAS Institute, NC, Cary, USA.

## Results

To validate the feasibility of the study, we sought the agreement of the participating hospitals.

In addition to the three centres involved in the pilot study, six other regional hospitals agreed to participate. The cohort was thus extended to the whole region.

We tested the process of providing patients with information. The patient leaflet was written according to CCTIRS recommendations [15].

To test this process, we gave the leaflet to ten patients and evaluated their understanding of it. This test helped us to improve and validate the contents and the format of the information leaflet.

We obtained approval from the regulatory authorities: at a national level, the protocol was successively approved by the CCTIRS and by the CNIL and at regional level, agreements were signed with each of the hospital management teams.

The availability and accessibility of the relevant variables in the medical files were reviewed.

The variables to be collected, which were initially decided by the steering committee, were in the patient files both during the inclusion period and during the follow-up consultations. At the end of the pilot phase, the list of variables was validated by the steering committee, and included in the CRF.

To validate the quality of the data obtained, the characteristics of patients included and non-included eligible patients were collected. These data were presented in Tables 1 and 2.

**Table 1:** Characteristics of patients at inclusion (in all 3 hospitals and for each participating hospital)

	Total 3 hospitals	Besançon	Mâcon	Belfort-Montbéliard
Number of patients	1849	599	476	774
Sex ratio M:F	1.46	1.45	2.13	1.17
Mean age in years	71.6	70.2	73.1	71.7
SD	+/− 12.9	+/− 13.4	+/−12.2	+/−12.9
[min – max]	[19 – 99]	[19 – 99]	[25 – 96]	[20–98]
Mean GFR (in ml/min/1.73 m2)	34.0	34.0	34.4	33.8
SD	+/− 12.5	+/− 12.9	+/−12.4	+/−12.3
[min – max]	[5 – 59.9]	[5.6 – 59.9]	[6.6 – 59.5]	[5 – 58.8]
GFR (in ml/min/1.73 m2)				
< 15	133 - 7.2%	45 - 7.5%	31 – 6.5%	57 – 7.4%
[15 – 30]	572 - 30.9%	193 - 32.2%	146 – 36.7%	233 -30.1%
[30 – 45]	730 - 39.5%	220 - 36.7%	186 -39.1%	324 – 41.9%
[45 – 60]	414 - 22.4%	141 - 23.5%	113 -23.7%	160 – 20.7%
Mean proteinuria (in g/24 h.)	1.0	1.1	1.1	1.0
SD	+/− 2.1	+/− 2.3	+/− 1.8	+/− 1.9
[min – max]	[0 – 26]	[0 – 26]	[0 – 13]	[0 – 20.8]
Diabetes	736 - 39.8%	220 - 36.8%	178 – 37.4%	338 – 43.7%
Type I	40 - 2.2%	25 - 4.2%	6 – 1.3%	9 - 1.2%
Type II	696 - 37.6%	195 - 32.6%	172 – 36.1%	329 - 42.5%
Hypertension	1634 - 88.4%	493 - 82.3%	451 – 94.7%	690 - 89.1%

**Table 2:** Comparison of characteristics of patients included vs non-included eligible patients

	Patients included *n* = 1849	Non-included eligible patients *n* = 51	*P*-value
Sex (n - %)			(K) *p* = 0.53
Male	1097 – 59.3%	28 – 54.9%	
Female	752 – 40.7%	23 – 45.1%	
Mean age in years	71.6	70.2	(S) *p* = 0.47
SD	+/− 12.9	+/− 14.1	
[min – max]	[19 – 99]	[21 – 99]	
Mean GFR (in ml/min/1.73 m2)	34.0	36.1	(S) *p* = 0.29
SD	+/− 12.5	+/− 13.0	
[min – max]	[5 – 59.9]	[14.3 – 59.1]	
GFR (in ml/min/1.73 m2)			(F) *p* = 0.75
< 15	133 - 7.2%	2 - 3.9%	
[15 – 30]	572 - 30.9%	16 - 31.4%	
[30 – 45]	730 - 39.5%	19 - 37.3%	
[45 – 60]	414 - 22.4%	14 - 27.5%	
Diabetes	736 - 39.8%	18 - 35.3%	(K) *p* = 0.52
Hypertension	1634 - 88.4%	40 - 78.4%	(K) *p* = 0.03

Table 1 presents the characteristics of patients at the time of inclusion.

Table 2 presents a comparison of the characteristics of patients included and non-included eligible patients.

Because this was an observational study which aimed to describe patient management, non-collected data were not considered to be missing data. However, the data deemed essential by the steering committee and which could have been defined as missing data were always accessible during the pilot study, except for the age of one patient and diastolic blood pressure for one patient. These data were as follows: for inclusion (age, sex, GFR, comorbidities, exposure to medication) and during follow-up (GFR, death, transplant, start of dialysis treatment, exposure to medication and onset of comorbidity).

The data on patients lost to follow-up are presented in Table 3.

**Table 3:** Outcome of patients included and lost to follow-up

Outcome of 1849 patients included	No. of patients
During follow-up	1686
. Including those not seen for at least 18 months with GFR > 45	302
Left study	159
. Including those who died	76
. Including those undergoing dialysis	68
. Including those receiving transplants	6
. Including those who changed hospitals	7
. Including those excluded for not meeting inclusion criteria	2
Possible lost to follow-up of a population of 1371 patients with GFR < 45 included prior to 31 March 2014 and not seen again for at least 18 months	4

Of the 1371 patients included before March 31^st^ 2014 with GFR < 45 ml/min/1.73 m^2^ and not seen at an appointment for at least 18 months, four patients were potentially lost to follow-up, i.e. 0.3%.

Of 1849 patients, 541 with a GFR < 60 ml/min/1.73 m^2^ had not been seen for an appointment for at least 12 months on 30 September 2014. These patients had either not yet attended an appointment, were possibly lost to follow up, or had been referred to their general practitioner. This figure falls to 8 for patients with a GFR < 45 ml/min/1.73 m^2^ not attending an appointment for at least 12 months.

The results of the quality control are presented in Table 4.

**Table 4:** Quality control

Hospitals	Number of controlled variables	% minor discrepancies	% major discrepancies
Besançon	297	3.70% (11)	0.34% (1)
Mâcon	562	2.31% (13)	0.36% (2)
Belfort-Montbéliard	444	3.15% (14)	0.45% (2)
Total 3 hospitals	1303	2.92% (38)	0.38% (5)

Check of quality of data gathered by CRAs at follow-up visits

The human resources required (number of CRAs) for data gathering in hospitals were calculated based on the pilot phase, taking into account differences in data entry time according to the availability of patient files in the hospitals, and the time required to ascertain the vital status of patients, and based on the possibility of extending the study to other hospitals.

Table 5 presents the human resources required (CRAs) and the estimated annual number of patients treated for renal insufficiency in each hospital.

**Table 5:** Human resources required (CRA) and annual number of patients estimated by the CRAs (patients treated for CKD in participating hospitals)

Hospitals	Human resources (equivalent of CRA days per week)	Annual number of patients estimated by the CRAs
Auxerre	1	500
Besançon	1.5	1000
Chalon sur Saône	1	500
Dijon	1.5	1000
Dôle	1	400
Mâcon	1	800
Belfort-Montbéliard	1.5	900
Sens	1	250
Vesoul	1	600
Total hospitals	10.5	5950

The exposure of patients at the time of inclusion to medication listed in the ANAES indicators [16] is presented in Table 6 (raw data are available in the Additional file 1).

**Table 6:** Patient exposure to the medication listed in ANAES indicators

Exposure of the 1849 patients	Number of patients/%
At least one ACE inhibitor	578/31.3%
At least one ARA II	570/30.8%
At least one ACE inhibitor or one ARA II	998/54.0%
At least one ACE inhibitor and one ARA II	75/4.1%
Metformine	115/6.2%
At least one NSAID	65/3.5%
Aminoglycoside	0/0%

## Discussion

The P-ND CRIS pilot study has enabled us to start the ND-CRIS study. We believe the experience gained during this pilot study must be shared and could assist other researchers who wish to implement major prospective cohorts in the field of chronic diseases such as CKD.

The feasibility, quality of data gathered and the evaluation of the resources required were taken into account in the design of the P-ND-CRIS pilot study.

As part of the feasibility assessment and before the pilot study was launched, a pre-test phase was realized to assess the willingness of nephrology departments across Bourgogne-Franche-Comté to participate in the study. In agreement with the nephrologists, this phase confirmed the need for CRAs to collect the data with secure access to electronic data. The involvement of the nephrologists was also considered necessary, particularly to validate the information collected by the CRAs. Without this initial review of the situation, the pilot phase would not have been possible.

As far as the regulatory applications and the patient information leaflet were concerned, the requirements vary in different countries. In France, approval is required from the CCTIRS to establish an observational cohort. The CCTIRS validates the patient information and acts as an ethics committee. Approval is also required from the CNIL, which ensures that the data collected remain anonymous.

The quality of the data collected is essential for any epidemiological study, because unreliable data cannot be compensated for in the statistical analysis. Data gathering cannot be enforced in an observational study. However, a cohort must contain variables and relevant outcomes if it is to be exploitable and informative.

In our experience, the pilot phase must allow sufficient time to evaluate the availability of data to be collected at inclusion and during follow-up. In order to have sufficient inclusion and follow-up data, the P-ND-CRIS study was conducted over a three year period. During this time, we checked for the presence, the quality and the accessibility of the relevant variables in the medical files, both during the inclusion period and for the follow-up consultations.

The early data in the pilot phase highlighted the need to add some essential variables (HbA1c levels, albuminaemia, urinary urea, and urinary sodium) to be collected prospectively during the extension phase. The absence of data on vaccination status, in particular for influenza and hepatitis B, led us to encourage practitioners to include this information in their files, in particular from CKD stage 3b. This enabled us to design and improve the CRF.

One of the aims of the ND-CRIS cohort was to carry out pharmacoepidemiological studies. It was therefore necessary that information on all medication prescribed to patients was collected, not simply those mentioned by the national recommendations [16]. Access to health insurance databases would be required to obtain this information. However, given the care taken by nephrologists regarding medication in renal insufficiency patients, we know that the information gathered in our pilot study is adequate. A request for “matching” between the ND-CRIS database and the health insurance databases will nonetheless be made to the relevant authorities.

The data concerning exposure to different medication were efficiently collected during the pilot study, and it showed that the P-ND-CRIS population was not prescribed nephrotoxic medication (aminoglycosides, metformin and NSAIDS in particular) as outlined in the ANAES guidelines [16]. However, the proportion of patients exposed to an ACE inhibitor or an ARA2 drug was 54%, which seems rather low given the number of subjects with hypertension (88.4%). This could be because this particular exposure was measured at inclusion, and not after follow-up started in the nephrology department. Finally, given the number of patients with hypertension <130/80 Hg (22.9%, results not shown) it is clear that great improvements can be made to achieve renal protection in line with the recommendations.

The quality assessment is an important element for epidemiological research because insufficiently reliable data can be detrimental to the analyses and affect conclusions in unpredictable ways.

The quality assessment conducted in the three hospitals on 1303 variables showed low rates of discrepancies between data in the CRFs and data in the medical files.

The pilot study should guarantee the representativeness of the target population. To do this, baseline characteristics of the non-included eligible population must be compared with those of patients included.

The descriptive data presented here do not provide evidence of any differences between eligible patients who were included and those who were not.

The P-ND-CRIS population, which was consistent across the three hospitals in the pilot study, is probably representative of the population of CKD patients followed up in the nephrology departments of the Bourgogne-Franche-Comté region, and probably in France as a whole. This national representativeness can be checked once we have the results of the national French cohort, CKD-Rein, which represents the whole country [17].

In a major longitudinal cohort, it seems that limiting the number of patients lost to follow-up is even more important than the rate of missing data in order to judge the quality of the data. If data gathering cannot be enforced in an observational study, we cannot consider all the non-entered variables to be missing data. However, it is essential that no data are missing with regards to the relevant outcomes, which is the case in our pilot study.

As far as the patients lost to follow-up are concerned, we should first define them. In a heterogeneous population of patients followed by a nephrologist with GFR <60 ml/min/1.73 m^2^, only patients who should have attended further appointments with the nephrologist can be considered as lost to follow-up. In France, general practitioners are encouraged to identify and follow patients with a GFR <60 ml/mn/1.73 m^2^. However, according to the recommendations, GPs only refer patients to nephrologists systematically in the event of GFR below 45 ml/min/1.73 m^2^ [13] or if there are specific problems. Thus, since our population only concerns patients followed by nephrologists, the ND-CRIS cohort cannot claim to represent patients at stage 3a. In addition, since only some of these patients are followed in nephrology departments, with consultations sometimes 18 months apart, they were not included in our definition of patients lost to follow-up. In our study, the rate of patients lost to follow-up (0.3%) was well below the threshold used as one of the quality criteria for CKD cohorts [18].

Strategies designed to reduce the numbers lost to follow-up should be assessed and implemented. Such strategies have been developed by other teams [19, 20]. Information should be gathered to characterize subjects lost to follow-up such as vital status, or by referring to birth and death registries.

Another important point for establishing a cohort effectively is to plan the resources required, particularly the CRAs. The main elements taken into account to estimate resources were the annual number of patients per hospital, the average time required to collect data per patient at inclusion and in follow-up appointments and the costs incurred for CRAs to travel to hospitals. We calculate that 10.5 CRA days are required per week to manage the ND-CRIS cohort in the 9 hospitals in the region.

## Conclusion

Without specific guidelines on conducting a pilot study before establishing a major prospective study, we believe our experience will prove useful for other researchers.

The P-ND-CRIS internal pilot study has demonstrated the feasibility of the ND-CRIS cohort. It has enabled us to plan and implement the ND-CRIS cohort, which should contribute to the epidemiological study of kidney disease in France, such as the REIN registry [21] and the CKD-Rein cohort [17]. An internal pilot study enables us to establish a cohort with a large number of patients and well-organized follow-up from the start.

The strengths of the P-ND CRIS study were the large sample of patients and the quality and accessibility of data, with very few patients lost to follow-up. We expect to recruit more than 5000 patients, which will, to our knowledge, make the ND-CRIS cohort one of the largest open prospective cohorts on pre-end stage CKD.

## Additional file

Additional file 1: Description of data: Inclusion worksheet: patients’ characteristics at inclusion. (XLSX 1568 kb)

## Abbreviations

ACE: Angiotensin converting enzyme inhibitor
ADELF: Association des Epidémiologistes de Langue Française
ADEREST: Association pour le Développement des Études et Recherches Épidémiologiques en Santé Travail
AEEMA: Association pour l’Etude de l’Epidémiologie des Maladies Animales
ANAES: Agence Nationale pour Accréditation et l’Evaluation des Soins
ARA2: Angiotensin 2 Renin Antagonist
CCTIRS: Advisory Committee for Data Processing in Health Research at the French Research Ministry (equivalent to an Ethics Committee)
CH: Hospital Centre
CHRU: Regional University Hospital Centre
CKD: Chronic kidney disease
CNIL: Comité National de l’Informatique et des Libertés (French data protection authority)
CRA: Clinical research associate
CRF: Case report form
CRI: Chronic renal insufficiency
DIM: Medical Information Department
EPIRAN: Epidémiologie de l’insuffisance renale dans l’agglomeration nanceienne
EPITER: Association pour le développement de l’épidémiologie de terrain
ESRD: End stage renal disease
GFR: Glomerular filtration rate
HAS: Haute Autorité de Santé (French National Authority for Health in charge of health technology assessment)
InVS: Institut National de Veille Sanitaire (French Institute for Public Health Surveillance)
ISPE: International Society for Pharmacoepidemiology
MDRD: Modification of diet in renal disease
ND-CRIS: Non-dialysis chronic renal insufficiency study
PHISQUARE: Public Health Impact Institute
